# Efficacy and safety of Ixekizumab *vs*. low-dose IL-2 *vs*. Colchicine *vs*. standard of care in the treatment of patients hospitalized with moderate-to-critical COVID-19: A pilot randomized clinical trial (STRUCK: Survival Trial Using Cytokine Inhibitors)

**DOI:** 10.1590/0037-8682-0565-2022

**Published:** 2023-04-14

**Authors:** Lívia Pimenta Bonifácio, Eduardo Ramacciotti, Leandro Barile Agati, Fernando Crivelenti Vilar, Anna Christina Tojal da Silva, Paulo Louzada, Benedito Antônio Lopes da Fonseca, Hayala Cristina Cavenague de Souza, Caroline Candida Carvalho de Oliveira, Valéria Cristina Resende Aguiar, Carlos Augusto de Aguiar Quadros, Cesar Dusilek, Kengi Itinose, Ricardo Risson, Lucas Roberto Rivabem Ferreira, Renato Delascio Lopes, Esper Georges Kallas, Fernando Bellissimo-Rodrigues

**Affiliations:** 1 Universidade de São Paulo, Faculdade de Medicina de Ribeirão Preto, Ribeirão Preto, SP, Brasil.; 2 Science Valley Research Institute, São Paulo, SP, Brasil.; 3 Grupo Leforte, Hospital e Maternidade Christóvão da Gama, Santo André, SP, Brasil.; 4 Hospital do Rocio, Campo Largo, PR, Brasil.; 5 Brazilian Clinical Research Institute, São Paulo, SP, Brasil.; 6 Duke University Medical Center - Duke Clinical Research Institute, Durham, North Carolina, USA.; 7 Universidade de São Paulo, Faculdade de Medicina, Departamento de Moléstias Infecciosas e Parasitárias, São Paulo, SP, Brasil.

**Keywords:** COVID-19, SARS-CoV-2, Cytokine storm, Ixekizumab, Interleukin-2, Colchicine

## Abstract

**Background:**

Cases of coronavirus disease 2019 (COVID-19) requiring hospitalization continue to appear in vulnerable populations, highlighting the importance of novel treatments. The hyperinflammatory response underlies the severity of the disease, and targeting this pathway may be useful. Herein, we tested whether immunomodulation focusing on interleukin (IL)-6, IL-17, and IL-2, could improve the clinical outcomes of patients admitted with COVID-19.

**Methods:**

This multicenter, open-label, prospective, randomized controlled trial was conducted in Brazil. Sixty hospitalized patients with moderate-to-critical COVID-19 received in addition to standard of care (SOC): IL-17 inhibitor (ixekizumab 80 mg SC/week) 1 dose every 4 weeks; low-dose IL-2 (1.5 million IU per day) for 7 days or until discharge; or indirect IL-6 inhibitor (colchicine) orally (0.5 mg) every 8 hours for 3 days, followed by 4 weeks at 0.5 mg 2x/day; or SOC alone. The primary outcome was accessed in the “per protocol” population as the proportion of patients with clinical improvement, defined as a decrease greater or equal to two points on the World Health Organization’s (WHO) seven-category ordinal scale by day 28.

**Results:**

All treatments were safe, and the efficacy outcomes did not differ significantly from those of SOC. Interestingly, in the colchicine group, all participants had an improvement of greater or equal to two points on the WHO seven-category ordinal scale and no deaths or patient deterioration were observed.

**Conclusions:**

Ixekizumab, colchicine, and IL-2 were demonstrated to be safe but ineffective for COVID-19 treatment. These results must be interpreted cautiously because of the limited sample size.

## INTRODUCTION

According to the World Health Organization (WHO), since the beginning of the coronavirus disease 2019 (COVID-19) pandemic, there have been approximately 620 million cases worldwide and 6,5 million deaths. Vaccination has significantly decreased the number of cases and hospitalizations, but there are still several barriers to global access to vaccines, which cause vulnerable populations to persist. Uncertainties concerning the duration of vaccine protection and the efficacy against emerging variants raise questions about the risk of infection in already vaccinated individuals. Collectively, these are sufficient reasons to justify the current need for novel treatments. Different therapeutics have been investigated and used to reduce the symptoms and treat COVID-19 including anticoagulants, antiviral drugs, antibiotics, monoclonal antibodies against SARS-CoV-2, systemic corticosteroids, and immunomodulators. The clinical guidelines for COVID-19 management are still being changed and adapted to include new evidence^1^.

A complex immune hyperactivation with consequent dysregulation of cytokine release, i.e. the “cytokine storm”, underlies the pathogenesis of COVID-19 and is characterized by systemic inflammation and multiorgan dysfunction. These include elevated blood levels of interleukin-1β (IL-1β), IL-6, tumor necrosis factor (TNF), interferon-γ (IFN-γ), IFN-induced protein 10 (IP-10), macrophage inflammatory protein (MIP) 1α and 1β, and vascular endothelial growth factor (VEGF)^2^. The increased cytokines can also enter the systemic circulation and cause extrapulmonary conditions, contributing to multiple organ dysfunctions^3^. In addition, lymphopenia with decreased numbers of CD4+ T cells, CD8+ T cells, and B cells is a key feature of COVID-19^4^. Therefore, targeting inflammatory signaling may be useful for the treatment of COVID-19.

IL-6 has been reported as a key contributor to disease complications and is a strong predictor of survival in COVID-19 patients^5^. Increased IL-6 levels are known to modulate the differentiation of pro-inflammatory IL-17-producing T helper cells (Th17) and to reduce the generation of regulatory T cells (Treg) which play significant roles in suppressing inflammation^6^. Increased Th17/IL-17 and decreased Treg have been reported in COVID-19 patients^7^ ^-^ ^11^. Importantly, the function of Treg is induced by low doses of IL-2, an anti-inflammatory cytokine^12^ whose signaling is suggested to be reduced in COVID-19 patients, making the manipulation of IL-2 levels possible in COVID-19 treatment^13^ ^,^ ^14^.

Crosstalk between IL-17 and IL-6 at the cell signaling level as IL-17 receptor activation increases the expression of IL-6 and can synergize with IL-6 in the induction of pro-inflammatory NF-kB signaling pathways^15^ ^,^ ^16^. Interestingly, direct activation of the IL-17 receptor by the SARS-CoV2 open reading frame 8 (ORF8) protein was suggested to contribute to cytokine storm in COVID-19 via increased expression of IL-6, TNF-alpha, IL-1β, and IL-12^17^. Inhibition of IL-17 is commonly used as a treatment strategy to reduce the number of lesions associated with inflammatory autoimmune diseases^18^ ^-^ ^20^. An example of such an inhibitory agent is ixekizumab, a humanized monoclonal antibody with high affinity to IL-17A, thus preventing interaction with the IL-17RA receptor and downstream signaling. ixekizumab has a quick onset of action, high tolerability, and well-established safety profile, and it is used for the treatment of severe psoriasis plaque, psoriatic arthritis, and axial spondyloarthritis^21^ ^,^ ^22^. This drug profile may be useful for COVID-19 treatment.

Owing to its central role in COVID-19 pathogenesis, IL-6 could be a very attractive target for Covid-19 immunomodulation therapies. The anti-IL-6 receptor monoclonal antibody tocilizumab, investigated in the RECOVERY trial, was shown to reduce 28-day mortality and invasive mechanical ventilation or death when compared to the standard of care (SOC) of hospitalized COVID-19 patients with hypoxia and systemic inflammation^23^. Similar effects were also found by REMAP-CAP, in which tocilizumab and sarilumab were similarly effective in improving survival and reducing the duration of organ support in critically ill patients compared to the usual treatments^24^. A beneficial effect of colchicine, an anti-inflammatory agent used to treat gout, viral pericarditis, and coronary heart disease^25^ ^-^ ^29^ could be expected in COVID-19 due to its indirect inhibition of IL-6. However, several trials including GRECCO^30^, COLCOVID^31^, RECOVERY^23^, COLCHIVID^32^, COLORIT^33^, and COLCORONA^28^ demonstrated, colchicine as a safe therapeutic but reported conflicting results about the effectiveness and beneficial effects on clinical improvement of COVID-19 patients. These studies are difficult to interpret because of differences among study protocols regarding the clinical characteristics of participants, concomitant use of medications, study design, and/or methodological limitations.

COVID-19 immunopathology and the results from earlier trials continue to make cytokine storm control a promising approach to treating disease; therefore, there is still a need for new evidence aiming to repurpose drugs used for autoimmune diseases as alternative treatments for COVID-19. The present study proposed that modulation of the inflammatory immune response via one of the following targets: IL-6, IL-17, and IL-2 could improve clinical outcomes of COVID-19 patients when compared to the SOC treatment.

We aimed to evaluate the efficacy and safety of ixekizumab (IL-17 inhibitor), low-dose IL-2, and colchicine (indirect IL-6 inhibitor) combined with SOC treatment in comparison with SOC alone for the treatment of hospitalized patients with moderate-to-critical COVID-19. 

## METHODS

### Trial design

This was a pragmatic, open-label, multicenter, prospective, randomized controlled trial in hospitalized patients with moderate-to-critical COVID-19. 

### Participants

Patients who required hospitalization for COVID-19 were invited to participate in the study at different hospital inpatient units. None of the participants had been vaccinated against COVID-19. The criteria for hospital admission and hospitalization in the ICU followed the recommendations of the São Paulo State Department of Health, Resolution SS-28 of March 17/2020, prepared by the Hospital das Clínicas of the School of Medicine of the University of São Paulo. The presence of an increased respiratory rate or desaturation (respiratory rate ≥24 IRPM and O_2_ saturation <93%) was the criterion for hospital admission. The presence of one of these conditions was considered a criterion for hospitalization in the ICU: no improvement in O_2_ saturation despite oxygen supply or arterial hypotension, changes in capillary filling time, changes in the level of consciousness, or oliguria.

### Inclusion criteria

- Male and nonpregnant female patients 18 years of age or older
- Positive reverse-transcriptase polymerase chain reaction (RT-PCR) assay for SARS-CoV-2 in respiratory tract samples
- Pneumonia confirmed by chest imaging

### Exclusion criteria

- Age <18 years
- The physician’s decision that involvement in the trial was not in the patient's best interest.
- Known severe liver disease (e.g., cirrhosis, alanine aminotransferase level >5× the upper limit of the normal range or aspartate aminotransferase level >5× the upper limit of the normal range)
- Pregnancy or breast-feeding
- Known HIV infection (without control)
- Presence of unstable cardiovascular diseases
- Known history of gastrointestinal bleeding, uncontrolled peptic or duodenal ulcer
- Known history of hemophilia or other bleeding disorders
- History of organ transplantation or congenital immunodeficiency

### Study setting

This multicenter study was conducted at five research centers in Brazil. All selected sites were tertiary hospitals, that is, highly complex hospitals with their own intensive care units and local access to laboratory and imaging tests. 

### Ethical Considerations

The STRUCK trial was conducted in accordance with ethical principles of human experimentation and with the Declaration of Helsinki revised in 2000. All sites received approval from the Institutional Review Board (CAAE 37880620.0.1001.5440), and the study was registered at ClinicalTrials.gov (NCT04724629). All patients or their legal representatives signed an informed consent form before the study procedure.

### Randomization and Interventions

All participants received SOC in accordance with the current local clinical practice for COVID-19 management, which could include supplementation with O_2_ ventilation, corticosteroids (dexamethasone, methylprednisolone, or prednisone), anticoagulants (enoxaparin, rivaroxaban, or non-fractioned heparin), and/or antibiotics (azithromycin, ceftriaxone, clarithromycin). In addition to the SOC treatment, the participants received the following:

1. IL-17 inhibitor (ixekizumab 80 mg SC) 1 dose every 4 weeks
2. Low Dose IL-2 (1.5 million IU per day) for 7 days or until discharge
3. Indirect IL-6 inhibitor (colchicine) orally 0.5 mg every 8 hours for 3 days, followed by 4 weeks 0.5 mg 2x/day

**FIGURE 1:**
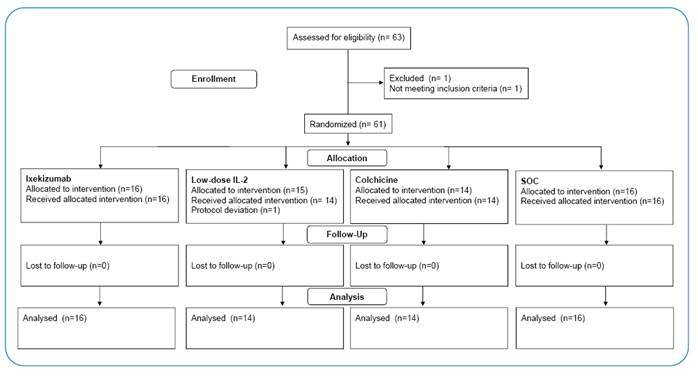
CONSORT Flow diagram of the study.

Randomization was performed in blocks of variable sizes facilitated by a central, concealed, web-based, automated system (RedCap, version 11.0.3). Enrolled participants were allocated (1:1:1:1) to receive ixekizumab, low-dose IL-2, indirect IL-6 inhibitor, or SOC alone. Figure 1 shows the design of the clinical trial.

### Visits and data collection

Data were collected via medical records and patient interviews entered into RedCap. Baseline measurements were defined as the clinical status assessment recorded on or before treatment initiation (randomization, study days 1 - D1). Daily visits until discharge were recorded for clinical assessments. The primary endpoint was assessed at the study visit on day 28 (D28). The participants had a follow-up visit 45 days after randomization (D45, telephone call). Plasma samples were collected on D1 and D28 to assess changes in the cytokine profile measured using a bead-based multiplex immunoassay for the simultaneous and quantitative detection of IFN-γ, IL-1β, IL-4, IL-6, IL-10, IL-18, and monocyte chemotactic protein (MCP1). 

### Outcomes

***Primary study outcome:*** The proportion of patients with clinical improvement, defined as a decrease of two points on the WHO’s seven-category ordinal scale for clinical improvement on D28 of the study^34^.

Seven categories of the Ordinal Scale for Clinical Improvement:

1. Non-hospitalized with the resumption of normal activities.
2. Non-hospitalized but unable to resume normal activities.
3. Hospitalized, without the need for supplemental oxygen.
4. Hospitalized, requiring supplemental oxygen.
5. Hospitalized, requiring high-flow nasal oxygen therapy, non-invasive mechanical ventilation, or both.
6. Hospitalized, requiring extracorporeal membrane oxygenation, invasive mechanical ventilation, or both.
7. Death.

### Secondary outcomes

1. The number of deaths accumulated on D28 of the study.
2. Changes in the cytokine profile on D28 of the study compared to baseline.
3. Quantitative assessment of treatment-related adverse effects.

### Statistics

The sample size of this pilot study was arbitrarily defined as 60 participants, estimating a minimum of 15 patients per arm to identify differences in the primary outcomes between the proposed treatments. This was a prospective randomized controlled trial with active control that tested the hypothesis of superiority, with a bilateral type I error rate of 0.05. The primary analysis was based on a “per protocol” population including all participants that were followed up through the last visit. The distribution of results according to severity was summarized as a percentage for each treatment arm. The baseline characteristics of each treatment arm were summarized. Descriptive measures such as mean, standard deviation (SD), and quartiles for quantitative variables and frequencies for categorical variables are presented. To compare the groups in relation to the outcome, binary analysis was performed, and the proportion of outcomes in each treatment arm was compared using Fisher's exact test. In addition, the relative risk was calculated with a 95% confidence interval. The patients’ evolution on the two-point scale was obtained by comparing their scores recorded at the time of screening and their last score recorded. A ≥2 point difference between the scores was considered an improvement. The Wilcoxon test for paired samples was used to assess the differences between plasma cytokine profiles at baseline D1 and D2). Analyses were performed using R 4.1.1 software^35^.

### Oversight and monitoring

The study was led by an academic steering committee comprising academic investigators from the research sites and other experienced clinical researchers who designed the trial. The Science Valley Research Institute (São Paulo, Brazil) was responsible for data and site management as well as for all statistical analyses. The Data Monitoring Board independently reviewed the data and safety of the study. 

## RESULTS

**FIGURE 2:**
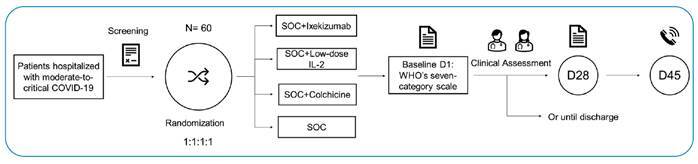
STRUCK Study design.

Between January 6, 2021, and July 9, 2021, 63 patients were assessed for eligibility. Of the total patients screened, 61 (96.8%) met the eligibility criteria and were enrolled and randomly assigned to receive either ixekizumab (n = 16 [25.8%]), low-dose IL-2 (n = 15 [24.6%]), colchicine (n = 14 [22.6%]), or SOC alone (n = 16 [25.8%]). Participants received the allocated intervention and were included in the “per-protocol” analysis. The flow diagram of the study is shown in Figure 2. The baseline characteristics of the 60 participants who received the allocated interventions are summarized in Table 1. The mean age was 48.9 years (SD 12.2); 23 (38.3%) were women, and 37 were men (61.7%). The mean body mass index was 31.7 kg/m² (SD 5.5); 27 (45.0%) had arterial hypertension, 13 (21.7%) had diabetes mellitus, 4 (6.7%) had respiratory disease, and 3 (5.0%) had heart disease. Regarding COVID-19 severity, 37 (61.7%) presented moderate disease (Score of 4 in the WHO 7-point ordinal clinical scale = participants hospitalized requiring supplemental oxygen) and 23 (38.3%) severe disease (Score 5 and 6 in the WHO scale = participants hospitalized requiring high-flow nasal oxygen therapy, non-invasive mechanical ventilation, or both; or requiring ECMO, invasive mechanical ventilation, or both), 8 (13.3%) were intubated, and 54 (90.0%) had a low risk of mortality as assessed by SOFA Score (Sequential Organ Failure Assessment; lower risk: score 0-6 points). The baseline demographic and clinical characteristics differed among the groups, probably because of the small sample size and block randomization. At baseline, the number of patients with severe disease and an increased risk of mortality (increased SOFA score) were higher in the ixekizumab group, the number of participants who were intubated was higher in the IL-2 group, and 100% of participants allocated to the colchicine group had a low risk of mortality (SOFA score 0-6).

**TABLE 1:** Baseline clinical characteristics of participants.

		Total	Ixekizumab	Low-dose IL-2	Colchicine	Standard of care
		N= 60	N = 16	N = 14	N = 14	N = 16
Age, years		48.9 (12.2)	48.5 (10.5)	45.4 (14.2)	56.2 (11.6)	46.1 (10.6)
BMI	Kg/m^2^	31.7 (5.5)	31.6 (6.0)	33.2 (6.3)	30.9 (5.8)	31.5 (4.2)
Sex	Female	23 (38.3)	5 (31.2)	9 (64.3)	4 (28.6)	5 (31.2)
	Male	37 (61.7)	11 (68.8)	5 (35.7)	10 (71.4))	11 (68.8)
Arterial hypertension		27 (45.0)	5 (31.2)	6 (42.9)	8 (57.1)	8 (50.0)
Diabetes mellitus		13 (21.7)	3 (18.8)	1 (7.1)	5 (35.7)	4 (25.0)
Respiratory disease		4 (6.7)	2 (12.5)	0 (0.0)	1 (7.1)	1 (6.2)
Heart disease		3 (5.0)	1 (6.2)	2 (14.3)	0 (0.0)	0 (0.0)
WHO Ordinal Scale	4. Hospitalized, requiring supplemental oxygen	37 (61.7)	7 (43.8)	10 (71.4)	8 (57.1)	12 (75.0)
	5. Hospitalized, requiring high flow nasal oxygen therapy, non-invasive mechanical ventilation, or both	17 (28.3)	7 (43.8)	1 (7.1)	6 (42.9)	3 (18.8)
	6. Hospitalized, requiring ECMO, invasive mechanical ventilation, or both	6 (10.0)	2 (12.5)	3 (21.4)	0 (0.0)	1 (6.2)
Intubation	Yes	8 (13.3)	2 (12.5)	5 (35.7)	1 (7.1)	1 (6.2)
SOFA Score	0-6	54 (90.0)	14 (87.6)	11 (78.6)	14(100.0)	15 (93.7)
	7-8	4 (6.6)	1 (6.2)	2 (14.3)	0 (0.0)	1 (6.3)
	10	1 (1.7)	1 (6.2)	0 (0.0)	0 (0.0)	0 (0.0)
	13	1 (1.7)	0 (0.0)	1 (7.1)	0 (0.0)	0 (0.0)

Data are presented as means (SD), n (%), or n/N (%). **BMI:** Body max index; **WHO Ordinal Scale:** World Health Organization Ordinal Scale for Clinical Improvement; **ECMO:** Extracorporeal Membrane Oxygenation; **SOFA:** Sequential Organ Failure Assessment.

The primary efficacy outcome (improvement in the WHO scale of ≥2 points) occurred in 14 (100%) participants assigned to the colchicine group, 13 (81.3 %) participants in the ixekizumab and SOC groups, and 9 (64.3%) participants in the low-dose IL-2 group (Table 2; p = 0.101). Seven (11.7%) participants in the study, three in the ixekizumab group, two in the low-dose IL-2 group, and two in the SOC group experienced clinical deterioration as assessed by changes in the WHO 7-point ordinal clinical scale on D28 in comparison to the baseline. None of the patients in the colchicine group presented clinical deterioration (Supplementary Table 1). Death occurred in eight (13.3%) participants in the study, with three in the ixekizumab group, three in the low-dose IL-2 group, and two in the SOC group, and there was no death in the colchicine group (Table 3; p = 0.340). The frequency of adverse events did not differ significantly between groups (Table 4; p = 0.489).

**TABLE 2:** Participants who experienced an improvement in clinical status at day 28, defined by a decrease greater or equal to two points on the World Health Organization’s seven-category ordinal scale for clinical improvement.

	N	%	Total	Relative risk (CI 95%)
Ixekizumab	13	81.3	16	1.00 (0.72 - 1.39)
Low-dose IL-2	9	64.3	14	0.79 (0.50 - 1.25)
Colchicine	14	100.0	14	1.23 (0.97 - 1.55)
SOC	13	81.3	16	1
**Total**	**49**	**81.7**	**60**	-

**SOC:** Standard of care; **IL-2:** interleukin 2. p-value Fisher's exact test: 0.101; **CI:** confidence interval.

**TABLE 3:** Survival rates on day 28.

	Survival on day 28					
	Survival		Mortality			
	N	%	N	%	Total	Relative risk (CI 95%)
Ixekizumab	13	81.3	3	18.8	16	1.50 (0.29-7.81)
Low-dose IL-2	11	78.6	3	21.4	14	1.71 (0.33-8.83)
Colchicine	14	100.0	0	0.0	14	-
SOC	14	87.5	2	12.5	16	1
**Total**	**52**	**86.7**	**8**	**13.3**	**60**	

**SOC:** Standard of care; **IL-2:** interleukin 2; **p-value Fisher's exact test:** 0.340; **CI:** confidence interval.

**TABLE 4:** Assessment of adverse events.

	N	%	Total
Ixekizumab	4	25.0	16
Low-dose IL-2	4	28.6	14
Colchicine	1	7.3	14
SOC	4	25.0	16
Total	13	21.7	60

**SOC:** Standard of care; **IL-2:** interleukin 2; **p-value Fisher's exact test:** 0.489; **CI:** confidence interval.

The cytokine profile assessed on D28 of the study did not differ significantly from the baseline (Supplementary Table 2). In the ixekizumab group, there was a reduction in the levels of pro-inflammatory cytokines IFN-γ (2.5 fold), IL1-β (46.7 fold), and IL-6 (4.3 fold), with elevation in the levels of pro-inflammatory cytokine IL-18 (1.5 fold), and the two anti-inflammatory cytokines IL-4 (2.7 fold) and IL-10 (4.6 fold), and a decrease in MCP-1 (1.1 fold). In the colchicine group, there was a reduction in the levels of the pro-inflammatory cytokines IFN-γ (1.4 fold), IL1-β (1.3 fold), IL-6 (8.3 fold), and IL-18 (1.0 fold), with a concomitant reduction of the anti-inflammatory cytokines IL-4 (1.3 fold) and IL-10 (4.0 fold) and MCP-1 level (1.1 fold). In the SOC group, there was a reduction in the levels of IFN-γ, IL1-β, IL-18, and MCP-1 by approximately 1.0 fold and IL-6 (3.0 fold) with a concomitant increase in the levels of IL-4 (2.0 fold), and IL-10 (1.0 fold).

## DISCUSSION

Mounting evidence has shown the role of a hyperinflammatory response in the severity of COVID-19, leading to multiorgan dysfunction and death. Targeting inflammatory signaling may be useful for treating COVID-19. Identifying novel treatments for COVID-19 may still have a significant impact on public health, especially in at-risk and socially vulnerable populations without access to vaccination. Herein, as a proof-of-concept, we tested whether the modulation of the inflammatory response via IL-6 (colchicine), IL-17 (ixekizumab), and low doses of IL-2 could improve clinical outcomes of moderate to critical COVID-19 in hospitalized patients. 

Our findings demonstrate that the use of ixekizumab, colchicine, or low doses of IL-2 is safe, as demonstrated by the low rate of treatment-related adverse events. Interestingly, changes in the cytokine profile were observed with all the treatments, with a decrease in pro-inflammatory cytokines, especially IL-6, and an increase in anti-inflammatory cytokines. Several studies evaluating the cytokine profiles of patients with moderate and severe disease present IL-6 as a predictive biomarker of disease deterioration^5^. However, the clinical effectiveness of the treatments evaluated in our study should be interpreted with caution. 

Colchicine is a promising treatment for patients with moderate-to-critical COVID-19 during their hospital stay. In the colchicine group, all participants showed an improvement of ≥2 points on the WHO 7-point Ordinal Scale (primary endpoint). No deaths or patient deterioration were observed in the colchicine group. We cannot rule out whether the results observed in the colchicine group were favored by randomization issues, which resulted in the allocation of participants with a lower risk of death to this group. Although a possible beneficial effect of colchicine is expected, considering its mechanism of action, conflicting results were found when the drug was used to treat patients with COVID-19. Pascual-Figál (2021), despite not reporting positive effects of colchicine on changes in the WHO 7-point ordinal clinical scale, found that clinical deterioration (+1 WHO scale at any time), need for ICU admission, and progression to mechanical ventilatory support were more frequent in the control group^36^. Tardif et al. (2021) reported that colchicine led to a lower rate of the composite of death and hospital admission among outpatients with a positive PCR test for SARS-CoV-2 when compared to the placebo group^28^ and Poudorwlat et al. (2022) found that colchicine treatment improved the percentage change of dyspnea according to the New York Heart Association guidelines when compared to the control group (32% versus 20% respectively)^37^, Cecconi et al. (2022) and Absálon-Aguiar et al. (2021) found no benefit of colchicine treatment in individuals with pneumonia or severe illness^32^ ^,^ ^38^.

In our study, treatment with ixekizumab did not improve patient recovery compared to the SOC. Anti-IL-17 monoclonal antibodies have been previously evaluated in patients with COVID-19. Netakimab treatment in patients with severe COVID-19 decreased inflammation (c-reactive protein (CRP) and IL-6) and significantly improved pulmonary structure and function, leading to reduced ICU requirement and mortality rate^39^. In the study by Avdeev et al., netakimab was also shown to ameliorate inflammation (decrease in blood CRP levels) and blood oxygenation in patients with severe COVID-19 despite no effects on the requirement of mechanical ventilation or mortality rate^40^. Interestingly, anti-IL-17 treatment with netakimab or secukinumab in patients with severe COVID-19 decreased the incidence of pulmonary embolism, suggesting that IL-17 is a key player contributing to this disease complication^40,^ ^41^ which is in line with the reported pro-coagulant and pro-thrombotic effects of IL-17^42^. The treatment of patients with moderate COVID-19 with netakimab significantly reduced systemic inflammation and improved clinical disease, decreasing the length of stay and mortality^43^. Altogether, these results suggest that anti-IL-17 treatment efficacy is dependent on the severity of the disease, being more effective in COVID-19 cases where the inflammation status is more intense.

A study conducted with patients with severe COVID-19 at the beginning of the pandemic demonstrated the potential of the use of low doses of IL-2 due to its immunomodulatory effect. In the treated group, there was a significant increase in the peripheral lymphocyte count when compared to the non-IL-2 group at discharge. Despite this finding, no differences were observed in the length of hospital stay. Considering that the population covered was very heterogeneous, with a relatively small number of patients tested, further tests are needed to ascertain the effects and safety of this treatment^44^.

The management and drug treatment of patients with COVID-19 who require hospitalization remains challenging. The Solidarity Trial, promoted by the WHO, to treat COVID-19 with antivirals did not have the expected positive impact, driving the international scientific community to seek new treatment alternatives for severely affected patients. Randomization with lopinavir, hydroxychloroquine, and interferon (IFN)-β1a was discontinued for futility; however, the study with remdesivir continued, and the final results showed no significant effect on the in-hospital mortality rate of patients with COVID-19 who were already being ventilated, with only a small effect against death and/or progression to ventilation in other hospitalized patients^45^.

One of the limitations of this study was the small sample size, which might have contributed to the allocation issues. The STRUCK trial was originally conceived as an adaptative study in which a single treatment that has been demonstrated to be more effective in the pilot study (4 arms) would be compared with SOC alone. However, when the pilot study was terminated, the number of hospitalizations in Brazil was dramatically reduced due to vaccination, indicating that a larger trial would not be feasible in a timely manner. Our findings shed light on the potential use of immunomodulators targeting IL-17, 1 L-6, and IL-2 signaling pathways and future trials with larger study populations properly designed to benefit populations with specific characteristics, such as immunocompromised and unvaccinated individuals are needed.

## CONCLUSION

In the present pilot study, we evaluated the potential of modulation of the IL-6 (colchicine), IL-17 (ixekizumab), and IL-2 signaling pathways to treat moderate to critical COVID-19 in hospitalized patients. Ixekizumab and IL-2 have been demonstrated to be safe but ineffective for COVID-19 treatment. Colchicine exhibited a statistically non-significant trend toward clinical improvement, but our findings need to be interpreted with caution because of our limited sample size and the differences observed in the baseline characteristics of the groups. 

## Appendix

**SUPPLEMENTARY TABLE1:** Changes in clinical status at day 28 in comparison to baseline assessed using the World Health Organization’s seven-category ordinal scale for clinical improvement.

	Total	Ixekizumab	Low-dose IL-2	Colchicine	Standard of care
	N= 60 (%)	N = 16 (%)	N = 14 (%)	N = 14 (%)	N = 16 (%)
3	4 (6.7)	0 (0.0)	2 (14.3)	0 (0.0)	2 (12.5)
2	2 (3.3)	2 (12.5)	0 (0.0)	0 (0.0)	0 (0.0)
1	1 (1.7)	1 (6,3)	0 (0.0)	0 (0.0)	0 (0.0)
0	2 (3.3)	0 (0.0)	2 (14.3)	0 (0.0)	0 (0.0)
-1	2 (3.3)	0 (0.0)	1 (7.1)	0 (0.0)	1 (6.3)
-2	6 (10.0)	2 (12.5)	1 (7.1)	2 (14.3)	1 (6.3)
-3	24 (40.0)	5 (31.3)	5 (35.7)	6 (42.9)	8 (50.0)
-4	15 (25.0)	5 (31.3)	1 (7.1)	6 (42.9)	3 (18.8)
-5	4 (6.7)	1 (6.3)	2 (14.3)	0 (0.0)	1 (6.3)

Data are presented as n (%) or n (%). **WHO Ordinal Scale:** World Health Organization Ordinal Scale for Clinical Improvement, respectively. Delta was calculated as the score assessed on day 28 minus the score assessed on day 1. Negative values indicate clinical improvement.

## Appendix

**SUPPLEMENTARY TABLE:** 2: Plasma cytokine profiles (pg/mL) in COVID-19 patients at baseline (D 1) versus day 28 (D 28).

	Groups	Ixekizumab	IL-2	Colchicine	SOC
**IFN- γ**	**D 1**	2.92 (1.81-74.19)	44.73 (4.13-85.33)	3.90 (2.21-17.91)	2.92 (1.90-8.65)
	**D 28**	(n=5)	(n=2)	(n=4)	(n=3)
		4.90 (1.56-25.17)	4.29 (3.68-4.90)	2.17 (1.20-13.77)	1.90 (0.81-10.57)
		(n=5)	(n=2)	(n=4)	(n=3)
	**p-value**	>0.999	n too small	0.2500	0.999
**IL-1 β**	**D1**	24.19 (10.82-64.50)	40.24 (5.46-75.01)	10.25 (1.73-38.49)	9.78 (1.31-51.44)
		(n=3)	(n=4)	(n=4)	(n=3)
	**D 28**	0.53 (0.53-1.06)	14.17 (6.47-21.87)	4.63 (0.71-31.73)	13.28 (1.96-37.09)
		(n=3)	(n=2)	(n-=4)	(n=3)
	**p-value**	0.2500	n too small	0.6250	>0.999
**IL-4**	**D 1**	1.94 (1.10-2.96)	2.38 (1.78-11.54)	2.63 (1.70-3.54)	0.97 (0.40-2.65)
		(n=7)	(n=3)	(n=6)	(n=6)
	**D 28**	1.49 (0.40-3.54)	2.31 (0.82-31.16)	1.10 (0.51-3.26)	2.07 (1.45-4.22)
		(n=7)	(n=3)	(n=6)	(n=6)
	**p-value**	0.2969	>0.9999	0.5625	0.4375
**IL-6**	**D 1**	23.93 (4.20-88.66)	4.21 (0.31-8.11)	4.83 (2.11-16.44)	2.39 (0.50-6.96)
		(n=5)	(n=2)	(n=4)	(n=4)
	**D 28**	7.72 (0.24-19.82)	0.63 (0.41-0.85)	0.76 (0.39-1.63)	1.02 (0.46-1.64)
		(n=5)	(n=2)	(n=4)	(n=4)
	**p-value**	0.3125	n too small	0.125	0.500
**IL-10**	**D 1**	12.96 (5.06-60.99)	5.22 (2.13-9.01)	7.57 (2.19-15.08)	3.80 (2.00-5.02)
		(n=5)	(n=3)	(n=4)	(n=5)
	**D 28**	2.08 (1.14-13.50)	7.36 (6.75-32.43)	1.69 (1.01-3.40)	3.99 (2.17-4.93)
		(n=5)	(n=3)	(n=4)	(n=5)
	**p-value**	0.0625	0.2500	0.1250	>0.9999
**IL-18**	**D 1**	385.20 (71.01-482.40)	139.10 (125.80-547.70)	234.50 (207.00-259.20)	185.30 (104.80-298.10)
		(n=7)	(n=3)	(n=6)	(n=6)
	**D 28**	366.60 (256.40-822.10)	284.90 (230.00-311.10)	231.20 (168.10-273.40)	131.50 (77.89-297.00)
		(n=7)	(n=3)	(n=6)	(n=6)
	**p-value**	0.4694	>0.9999	0.8438	0.8438
**MCP-1**	**D 1**	295.80 (139.10-361.70)	199.40 (174.40-224.30)	292.10 (213.80-338.10)	178.90 (159.00-333.90)
		(n=7)	(n=2)	(n=6)	(n=5)
	**D 28**	303.30 (224.30-661.40)	216.90 (197.80-235.90)	235.60 (158.60-354.10)	230.00 (119.20-238.90)
		(n=7)	(n=2)	(n=6)	(n=5)
	**p-value**	0.3750	n too small	0.3125	0.4375

Data are presented as the Median and Interquartile Range (IQR). Comparisons between baseline (D1) and D28 using Wilcoxon matched-pairs rank test, p>0.05. It was not possible to analyze cytokine values ​​from patients treated with low doses of IL-2 because of the small sample size.
